# Effects of Dance-Based Aerobic Training on Functional Capacity and Risk of Falls in Older Adults with Mild Cognitive Impairment

**DOI:** 10.3390/jcm14165900

**Published:** 2025-08-21

**Authors:** Marcelina Sánchez-Alcalá, María del Carmen Carcelén-Fraile, Paulino Vico-Rodríguez, Marta Cano-Orihuela, María del Mar Carcelén-Fraile

**Affiliations:** 1 Department of Health Sciences, Faculty of Health Sciences, University of Jaén, 23071 Jaén, Spain; 2 Department of Educational Sciences, Faculty of Social Sciences, University of Atlántico Medio, 35017 Las Palmas de Gran Canaria, Spain; 3 Asociación Científica Internacional Sobre la Innovación en Educación y Salud (ACIINES), 23007 Jaén, Spain; 4 Department of Health Sciences, Faculty of Health Sciences, University of Atlántico Medio, 35017 Las Palmas de Gran Canaria, Spain

**Keywords:** mild cognitive impairment, balance, flexibility, risk of falls, dance, aerobic training

## Abstract

**Background:** Older adults with mild cognitive impairment are at increased risk for physical decline and falls due to decreased strength, flexibility, balance, and gait. Dance-based aerobic training has emerged as a promising and enjoyable intervention to promote physical function and cognitive stimulation. This study aimed to evaluate the efficacy of a 12-week structured dance-based aerobic program, based on line dancing and Latin rhythms (e.g., salsa, merengue, and bachata), in improving functional capacity and reducing the risk of falls in older adults with mild cognitive impairment. **Methods**: A randomized controlled trial was conducted with 92 participants aged ≥65 years diagnosed with mild cognitive impairment. The participants were randomly assigned to an experimental group (dance-based training, twice weekly for 12 weeks) or a control group (usual activity). Outcomes included muscle strength (grip dynamometry), flexibility (back scratch and chair sit-and-reach tests), gait speed (Timed Up and Go test), balance (Tinetti scale), and total falls risk score (Tinetti). Mixed ANOVA and Cohen’s d were used for statistical analysis. **Results**: Significant improvements were observed in the experimental group on all variables compared to the control group. Muscle strength (*p* < 0.001, d = 0.86), gait speed (*p* = 0.026, d = 0.48), and upper and lower extremity flexibility (d = 0.43–0.79) improved significantly. The balance and gait components of the Tinetti scale also increased (*p* = 0.007 and *p* = 0.048, respectively), as did the total Tinetti score (*p* = 0.002, d = 0.67), indicating a reduction in the risk of falls. **Conclusions**: These findings suggest that, under structured conditions, dance-based aerobic training may serve as a promising non-pharmacological strategy to support healthy aging in older adults with mild cognitive impairment, although further validation in larger cohorts is needed.

## 1. Introduction

Population aging is a growing global phenomenon that presents significant health, social, and economic challenges [1]. According to the World Health Organization, the number of people over 60 years of age is expected to exceed two billion by 2050 [2]. This demographic shift demands effective strategies to promote active, independent, and healthy aging. Aging-related physiological changes, such as musculoskeletal deterioration and neurological impairments, can hinder daily functioning and quality of life in older adults [3,4].

One of the most prevalent conditions among older adults is mild cognitive impairment (MCI), a transitional state between normal aging and dementia [5]. MCI is associated not only with cognitive decline but also with physical deterioration, including reduced strength, balance, gait stability, and flexibility, factors that collectively increase fall risk [6]. Loss of muscle mass and strength, or sarcopenia, is especially relevant, as it undermines mobility and independence [7,8]. Research suggests that physical exercise can mitigate both motor and cognitive decline, particularly in people with MCI, by enhancing neuromuscular function and modulating neurobiological processes involved in cognition [9,10]. Flexibility and balance also decline with age, contributing to impaired mobility and greater susceptibility to falls [11]. Falls are among the leading causes of injury, hospitalization, and loss of autonomy in the elderly, especially in those with cognitive impairment [12].

In addition to the direct physical consequences, such as fractures and head injuries, falls can have significant psychological repercussions, such as the fear of falling again, which further reduces mobility and fosters a vicious cycle of sedentary lifestyle, weakness, and dependency [13]. It is estimated that approximately one-third of older adults fall at least once a year, and for those with cognitive impairment, this rate may be even higher. Therefore, fall prevention has become a priority objective within public health policies aimed at the aging population [14].

In this context, physical exercise is presented as an effective non-pharmacological strategy to improve both the physical and cognitive capacity of older adults [15]. Specifically, multicomponent exercise programs that integrate aerobic, strength, flexibility, balance, and coordination training have demonstrated benefits in improving executive function, processing speed, and overall well-being [16]. Among these interventions, structured dance programs have gained attention due to their potential to stimulate both motor and cognitive functions simultaneously. In this study, the intervention consisted of instructor-led sessions based on individual line dancing routines, without the need for a partner. The participants performed synchronized movements using styles such as salsa, merengue, bachata, pop, and rock. The dance classes followed a progressive structure, with increasing choreographic complexity adapted to the participants’ abilities. This distinction is important, as structured dance sessions differ significantly from spontaneous or recreational dancing by providing consistent cognitive–motor challenges. Prior research has demonstrated the cognitive and neural benefits of structured dance in older adults [17].

Dance-based aerobic training has been shown to improve physical fitness, working memory, mobility, and fall prevention. Among these interventions, dance-based aerobic training has gained growing interest due to its playful, social, and stimulating nature, which promotes adherence and engagement among participants [18]. Dance combines coordinated rhythmic movements, musical stimuli, and choreographic patterns that involve both physical and cognitive activation, making it a particularly promising tool for people with MCI [19]. Several studies have reported that dance not only improves general physical fitness but can also induce beneficial changes in the central nervous system, promoting brain plasticity, improving functional connectivity, and modulating neurotrophic factors, such as the BDNF (brain-derived neurotrophic factor). In this sense, line dancing, a dance style characterized by sequences of repetitive and synchronized steps performed in a line, stands out for its accessibility, ease of learning, and ability to simultaneously stimulate motor and cognitive functions [20,21]. Its progressive structure allows the complexity of the choreography to be adapted to the participants’ level, encouraging active participation and continuous learning [22].

Although dance-based interventions have shown promise in improving physical and cognitive outcomes in older adults, current evidence presents several important limitations. Most existing studies have focused on healthy older adults, with relatively few trials targeting individuals with mild cognitive impairment (MCI), despite their heightened risk of cognitive and functional decline [23,24]. Furthermore, previous interventions have often relied on isolated outcome measures, failing to capture the multidimensional effects that dance-based training may exert on strength, flexibility, gait, balance, and fall risk [25]. Meta-analyses have also reported high heterogeneity across studies, with considerable variability in sample characteristics, duration, intensity, and outcome domains [26]. While there is growing support for the cognitive benefits of dance in MCI, findings remain inconsistent, particularly regarding improvements in executive function, mood, and quality of life [27,28]. These limitations highlight the need for further research evaluating structured, choreographed dance interventions using multicomponent outcome measures in this vulnerable population. Therefore, the objective of this study was to analyze the effectiveness of a 12-week dance-based aerobic training program in improving functional capacity and reducing the risk of falls in older adults with mild cognitive impairment. Specifically, parameters such as muscle strength, flexibility, balance, gait speed, and total score on the Tinetti scale were evaluated to determine the impact of the intervention on key indicators of physical function and fall prevention in this vulnerable population. Based on previous research, we hypothesized that, compared to a control group engaged in usual activities, the participants in the dance-based intervention group would show greater improvements in (1) muscle strength, (2) flexibility of the upper and lower limbs, (3) balance, (4) gait speed, and (5) overall fall risk, as measured by standardized functional tests

## 2. Materials and Methods

### 2.1. Study Design and Participants

This study followed a randomized and controlled clinical trial design. Prior to initiating the intervention, all participants received comprehensive information about this research and provided written informed consent. The study protocol received approval from the Ethics Committee at the University of the Mid-Atlantic (CEI/05-007). All procedures adhered to the ethical standards outlined by the World Medical Association in the Declaration of Helsinki, which governs research involving human subjects. Furthermore, the trial was officially registered on ClinicalTrials.gov with the identifier NCT06130878.

**Figure 1:**
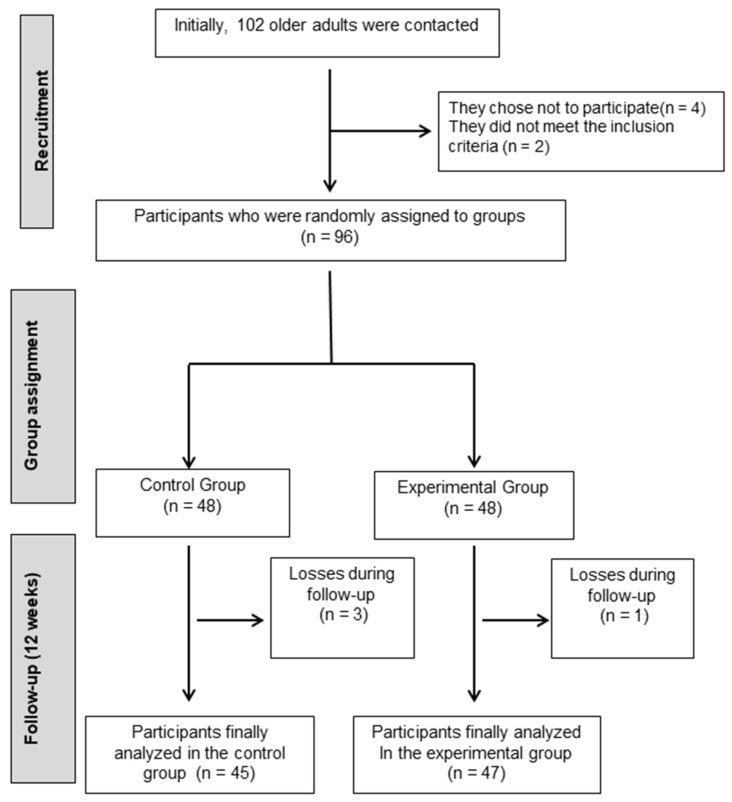
Participant flowchart.

Initially, 102 older adults diagnosed with mild cognitive impairment were approached to participate. Following the eligibility assessment, four participants did not complete the intervention: one from the experimental group and three from the control group. The reasons for withdrawal included personal scheduling conflicts (n = 2) and loss of interest (n = 2). None of the dropouts were related to adverse effects or health issues. A comparison of baseline characteristics between completers and non-completers revealed no substantial differences in age, sex distribution, or functional scores, suggesting minimal impact on group equivalence or internal validity. Consequently, a total of 96 participants were successfully enrolled and randomly divided into two groups, as illustrated in Figure 1.

#### 2.1.1. Inclusion Criteria

To be included in this study, participants had to be at least 65 years old and not currently engaged in any regular physical exercise programs. They were required to score below 24 on the Mini-Mental State Examination (MMSE), demonstrate sufficient physical independence to take part in the exercise sessions, and be able to understand the instructions and procedures involved in this study. Additionally, participants needed to provide written informed consent and attend more than 90 percent of the intervention sessions.

#### 2.1.2. Exclusion Criteria

Participants were excluded if they had any systemic condition, such as neurodegenerative, musculoskeletal, or visual disorders, that could interfere with their ability to complete the balance assessments or participate in physical activities. Individuals with vestibular diseases or disorders were also excluded, as well as those taking medications known to affect the central nervous system, balance, or coordination, including antidepressants, anxiolytics, or vestibular suppressants.

### 2.2. Sample Calculation

The Epidat statistical program was used to calculate the sample size. For the strength variable, the sample size was estimated with a 95% confidence interval and 90% statistical power. Expecting a mean difference of 0.57, based on the results of Mattle et al. [29], 76 people were estimated to be necessary. Given this, a 20% loss to follow-up rate was considered, resulting in a sample of 91 people. This variable was chosen because handgrip strength is a well-established biomarker of overall muscle strength and a strong predictor of adverse outcomes in older adults, including mobility limitation, disability, increased risk of falls, frailty, and mortality.

### 2.3. Randomization

The older adults who qualified for this study were randomly divided into two equally sized groups: an experimental group and a control group, following a 1:1 allocation ratio. This randomization process was conducted using a computer-generated table of random numbers. Group assignment was managed through sealed, opaque envelopes and handled by an independent party not involved in participant selection, intervention delivery, or data analysis. A total of 48 participants were placed in the experimental group (EG), where they took part in a dance-based aerobic training program, while the remaining 48 participants were allocated to the control group (CG). Those in the control group were instructed to maintain their usual daily activities without enrolling in any structured exercise programs. They also received general advice encouraging them to stay physically active.

### 2.4. Intervention

The aerobic dance-based training program was implemented over 12 weeks, with participants attending two 60 min sessions per week, totaling 24 sessions. All sessions were conducted in person at the facilities of the University of Atlántico Medio and were supervised by a certified and experienced instructor. The instructor guided participants safely and effectively, adapting choreography to individual abilities, and demonstrated each new routine from the front of the group, followed by a breakdown of each movement to ensure correct execution.

Each session was divided into three phases: (i) Warm-up (10 min): this phase involved low-intensity movements focused on stretching and flexibility, performed at a slow pace to facilitate adaptation to rhythm and coordination; (ii) Main phase (40 min): This segment consisted of five choreographed dance routines, each lasting approximately 4 min, with 2 min recovery breaks between routines to allow for rest, hydration, and preparation for the next dance. The routines included continuous lower-limb and torso movements, along with intermittent upper-limb movements, such as flexion–extension, abduction–adduction, lateral stepping, rotations, rhythm changes, forward and backward steps, heel raises, and arm or leg lifts. Choreographies were carefully sequenced to ensure progressive technical complexity across sessions. Musical genres used included salsa, merengue, bachata, rumba, pop, and rock. Songs were selected based on their cultural familiarity, lyrical content, and suitability for accompanying coordinated body movements, aiming to enhance rhythm, memory, and participant enjoyment. The approximate distribution of dance styles throughout the program was: merengue (25%), salsa (20%), bachata (15%), pop (15%), rock (15%), and rumba (10%). Weeks 1–4: Basic steps and simple rhythmic patterns were introduced, focusing on lateral, forward, and backward movement. Weeks 5–8: Choreographies increased in complexity, incorporating new steps and combinations, including turns that required enhanced coordination and sequential memory. Weeks 9–12: Advanced choreographies were introduced, involving complex movement sequences that participants were asked to perform without verbal prompts from the instructor, promoting cognitive recall and motor planning; and (iii) Cool-down (10 min): the session concluded with gentle stretching exercises performed to relaxing music, allowing participants to transition into a calm state.

### 2.5. Outcomes

All data and study variables were gathered by a researcher who was independent of both the group assignment process and the intervention itself. This individual was responsible for collecting the data at two time points: prior to group allocation and again at the conclusion of the intervention. The information obtained included both sociodemographic and clinical variables. Among the demographic details collected were age, marital status (categorized as married, single, separated/divorced, or widowed), employment status (retired, unemployed, or employed), and level of education (ranging from no formal education to university-level studies). Clinical measurements included body weight, recorded using a highly accurate Tefal digital scale (with a range of 100 g to 130 kg), and height, measured with an Asimed T201-T4 stadiometer. Additional anthropometric data were collected, such as waist circumference, measured at the narrowest point of the torso (typically around the navel), and hip circumference, assessed at the widest part of the hips and buttocks. From these values, the waist-to-hip ratio was calculated by dividing the waist measurement by the hip measurement. Body mass index (BMI) was also determined by dividing each participant’s weight in kilograms by the square of their height in meters (kg/m^2^).

#### 2.5.1. Muscle Strength

Muscle strength was assessed before and after the 12-week intervention using a handgrip dynamometer (model TKK 5001, Grip-A, Takei, Tokyo, Japan) equipped with a 4.5 cm handle. The participants were instructed to perform three maximal grip efforts with each hand, starting with the left and followed by the right, with a 30 s rest period between each attempt [30]. The highest value recorded across the trials was taken as the individual’s grip strength. A measurement below 20 kg was classified as indicative of reduced muscle strength [31].

#### 2.5.2. Flexibility

Functional flexibility was evaluated at baseline and at the end of the intervention using two specific tests: the back scratch test (BST) for the upper limbs [32] and the chair sit-and-reach test (CSRT) for the lower limbs [33]. The BST assessed the flexibility of the shoulder joints. The participants performed the test while standing, placing one arm over the shoulder and down the spine, while the opposite arm reached upward from the lower back. The test was then repeated using the opposite arms. The distance between the tips of the middle fingers of both hands was measured. If the fingers just touched, a score of zero was recorded. When the fingers did not meet, the gap was measured in centimeters and recorded as a negative value, while overlapping fingers resulted in a positive score. To measure flexibility in the lower extremities, particularly the hamstrings, the CSRT was used. The participants sat on a chair positioned against a wall for support and attempted to reach toward the toes of each foot—right and left. If their fingertips touched their toes exactly, a score of zero was given. Distances short of the toes were recorded as negative values, and those exceeding the toes were measured and noted as positive values, in centimeters.

#### 2.5.3. Balance

The Tinetti scale [34,35] evaluates both balance and gait throughout both pre- and post-intervention, with 16 items divided into two main sections. The first 9 items are dedicated to assessing balance, while the remaining 7 focus on gait performance. The final score is obtained by adding the points from both sections, with balance contributing up to 16 points and gait up to 12, resulting in a maximum total score of 28. A total score of 18 or below reflects a high risk of falling, scores between 19 and 24 suggest a moderate risk, and scores of 24 or higher indicate a low risk of falls. Both subscale scores (balance (0–16) and gait (0–12), in addition to the total TPOMA score (0–28), were analyzed separately.

#### 2.5.4. Gait Speed

Gait speed was assessed pre- and post-intervention using the Timed Up and Go (TUG) test [36]. This test consists of rising from a chair, walking a distance of three meters, turning around, and returning to a sitting position. The time recorded during the TUG test was converted to an estimate of walking speed using the formula [6/(TUG time)]*1.62, as validated by Martínez-Ramírez [37], who demonstrated a strong correlation between this derived value and directly measured gait speed in community-dwelling older adults. This method enables gait speed estimation without the need for a traditional walk test, making it suitable for clinical and field settings. A value ≤ 0.8 m per second [30] was considered the standard limit for a slow walking speed.

### 2.6. Statistical Analysis

All statistical procedures for this doctoral research were conducted using SPSS software, version 20.0 for Windows (SPSS Inc., Chicago, IL, USA). A significance level of *p* < 0.05 was applied for all analyses. Continuous variables were described using means and standard deviations, while categorical variables were summarized with frequencies and percentages. To verify whether the data followed a normal distribution, the Kolmogorov–Smirnov test was employed. In cases where normality was not confirmed, non-parametric tests (such as the Mann–Whitney U test or Wilcoxon signed-rank test) were applied accordingly to ensure statistical robustness. Baseline comparisons between the two groups were conducted using the Student’s *t*-test for continuous variables and the chi-square test for categorical ones. To evaluate differences over time and between groups, a mixed-design analysis of variance (ANOVA) was used. In this model, the group (control group vs. experimental group) was treated as the between-subjects factor, and the time point (pre- and post-intervention) as the within-subjects factor. The dependent variables included muscle strength (assessed via handgrip dynamometry), flexibility (measured by the back scratch test and chair sit-and-reach test), balance (evaluated using the Tinetti scale), and gait speed (measured with the Timed Up and Go test). Each dependent variable was analyzed separately, and interactions between group and time were explored. The effect size for both within- and between-group comparisons was calculated using Cohen’s d. According to established criteria, a d value below 0.2 indicates a negligible effect, values between 0.2 and 0.5 represent a small effect, between 0.5 and 0.8 a moderate effect, and values equal to or greater than 0.8 denote a large effect.

## 3. Results

In this study, 36.96% of the participants were male and 63.04% were female. The average age was 71.83 years, with a standard deviation of 2.96. The majority of individuals were retired (45.6%), married (35.4%), and had attained primary-level education (36.7%) (Table 1). When comparing the groups, no statistically significant differences were found in any of the sociodemographic variables. This baseline homogeneity between groups strengthens the internal validity of this study by reducing the likelihood that post-intervention differences are due to demographic confounding factors.

### 3.1. Muscle Strength

**Figure 2:**
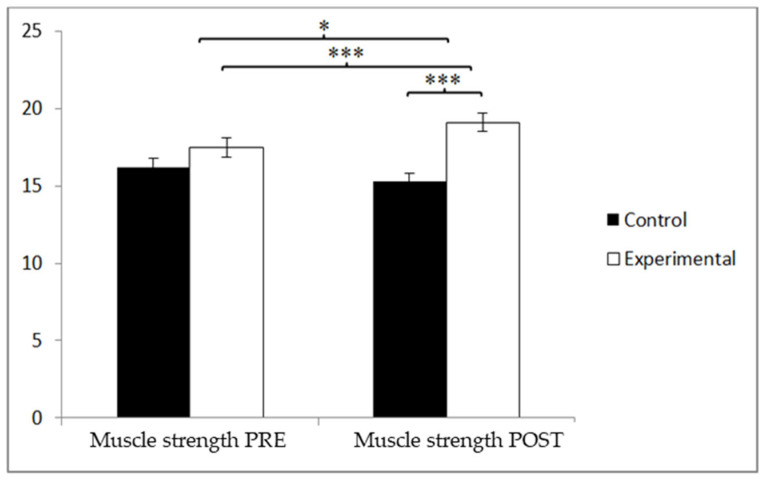
Between-group and within-group comparisons of muscle strength. * *p* < 0.05. *** *p* < 0.001.

Regarding muscle strength, the participants in the experimental group (EG) demonstrated higher values than those in the control group (CG) both before the intervention (17.22 ± 3.88 vs. 16.35 ± 3.84) and after it (18.97 ± 3.56 vs. 15.73 ± 3.90). Significant effects were found for the Group x Time interaction, F(1, 90) = 21.489, *p* = 0.000, and η^2^ = 0.193; for the Group factor, F(1, 90) = 7.558, *p* = 0.007, and η^2^ = 0.077; and for the Time factor, F(1, 90) = 4.857, *p* = 0.030, and η^2^ = 0.051 (Figure 2). A detailed analysis of the interaction effect revealed a statistically significant difference between the groups at the post-intervention stage, t(90) = −4.171 and *p* = 0.000, with a large effect size (Cohen’s d = 0.86). Additionally, a significant improvement in muscle strength was observed within the experimental group when comparing pre- and post-intervention measurements, t(46) = −8.724 and *p* = 0.000, although the effect size was small (d = 0.47). This difference in effect size reflects the fact that intergroup comparisons capture the contrast between divergent trajectories in both groups, while intragroup comparisons focus solely on the magnitude of change within a single group.

### 3.2. Gait Speed

**Figure 3:**
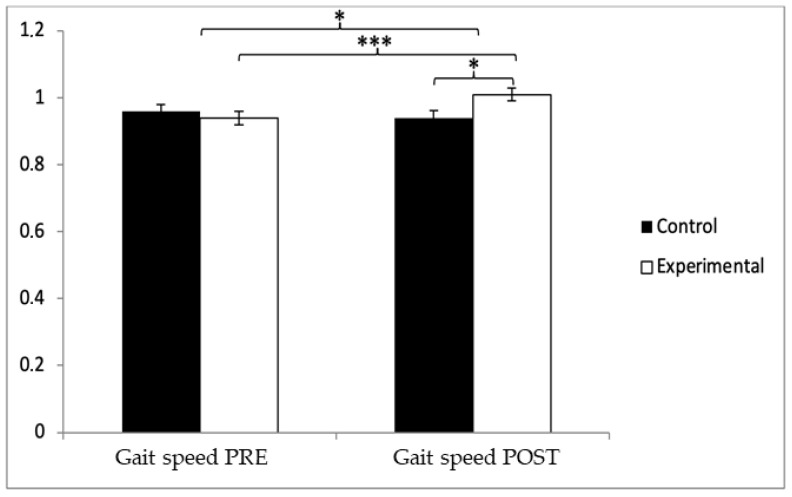
Between-group and within-group comparisons of gait speed. * *p* < 0.05. *** *p* < 0.001.

In terms of gait speed, the participants in the control group (CG) initially showed slightly higher values (0.96 ± 0.13) compared to those in the experimental group (EG), who averaged 0.94 ± 0.38 prior to the intervention. However, this trend reversed following the intervention, with the EG reaching a mean of 1.01 ± 0.17, surpassing the CG, which maintained a value of 0.94 ± 0.12. A significant Group x Time interaction was found, F(1, 90) = 15.491, *p* = 0.000, and η^2^ = 0.147, along with a significant main effect for Time, F(1, 90) = 4.910, *p* = 0.029, and η^2^ = 0.052. No significant differences were observed for the Group factor alone, F(1, 90) = 0.911, *p* = 0.342, and η^2^ = 0.010 (Figure 3). Although no significant main effect was found for the Group factor alone, the significant interaction and time effects indicate that the observed improvements in gait speed were primarily driven by temporal changes within the experimental group. This supports the effectiveness of the intervention over time rather than inherent baseline differences between groups. Further analysis of the interaction revealed statistically significant post-intervention differences between the two groups, t(90) = −2.267 and *p* = 0.026, with a small effect size (Cohen’s d = 0.48). Additionally, within the experimental group, a significant improvement was noted when comparing pre- and post-intervention gait speed, t(46) = −4.362 and *p* = 0.000, corresponding to a medium effect size (d = 0.41).

### 3.3. Flexibility

**Figure 4:**
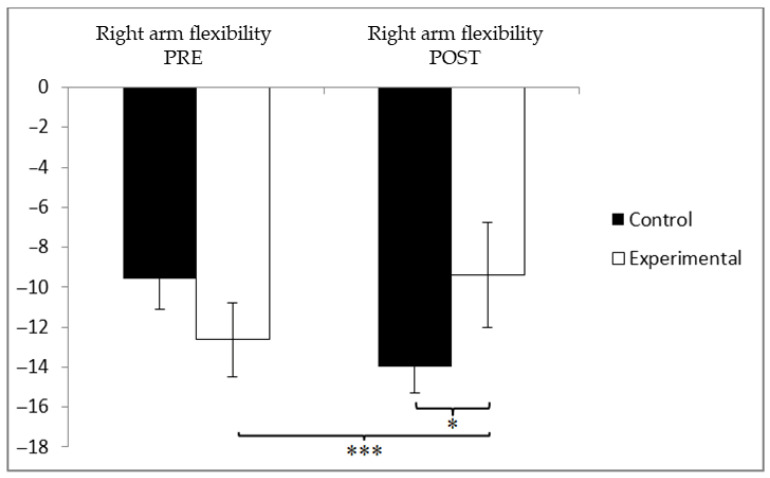
Between-group and within-group comparisons of right arm flexibility. * *p* < 0.05. *** *p* < 0.001.

In the right arm flexibility assessment, the participants in the experimental group (EG) initially showed lower values (−12.49 ± 10.22) compared to those in the control group (CG), who recorded −10.40 ± 11.90 before the intervention. However, after the intervention, the CG showed greater negative values (−14.36 ± 16.26) than the EG (−8.13 ± 7.15). This reduction, although not dramatic, is consistent with expected patterns in joint range of motion over time in sedentary older adults. A significant interaction effect between Group and Time was found, F(1, 90) = 12.003, *p* = 0.001, and η^2^ = 0.118, while no significant main effects were observed for Time, F(1, 90) = 0.029, *p* = 0.886, and η^2^ = 0.000, or for Group, F(1, 90) = 1.751, *p* = 0.338, and η^2^ = 0.010 (Figure 4). Further analysis of the interaction revealed statistically significant differences between the two groups at the post-intervention stage, t(90) = −2.396 and *p* = 0.019, with a medium effect size (Cohen’s d = 0.50). Moreover, within the experimental group, a significant improvement was observed when comparing pre- and post-intervention flexibility scores, t(46) = −5.654 and *p* = 0.000, corresponding to a small effect size (d = 0.49).

**Figure 5:**
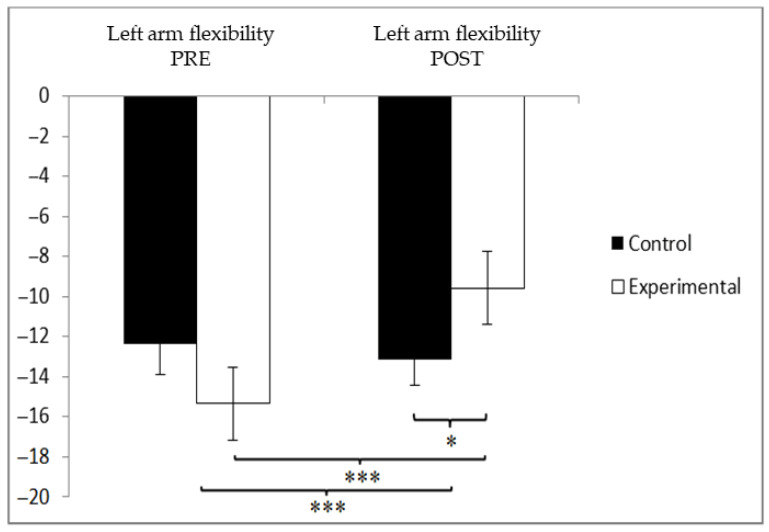
Between-group and within-group comparisons of left arm flexibility. * *p* < 0.05. *** *p* < 0.001.

For the left arm flexibility assessment, the experimental group (EG) initially demonstrated lower scores (−15.36 ± 9.71) compared to the control group (CG), which recorded −12.71 ± 12.00. However, following the intervention, this pattern reversed, with the CG obtaining lower values (−13.38 ± 12.10) than the EG (−8.79 ± 6.58). A significant interaction effect was observed between Group and Time, F(1, 90) = 54.149, *p* = 0.000, and η^2^ = 0.376, as well as a significant main effect for Time, F(1, 90) = 36.044, *p* = 0.000, and η^2^ = 0.286. No significant main effect was found for Group, F(1, 90) = 0.215, *p* = 0.644, and η^2^ = 0.002 (Figure 5). Further analysis of the interaction revealed significant post-intervention differences between the two groups, t(90) = −2.273 and *p* = 0.025, with a small effect size (Cohen’s d = 0.47). Additionally, within the experimental group, a statistically significant improvement was found between pre- and post-intervention scores, t(46) = −8.097 and *p* = 0.000, with a large effect size (d = 0.79). This data suggests a significant functional gain, potentially improving participants’ ability to perform daily tasks such as dressing, grooming, or reaching overhead.

**Figure 6:**
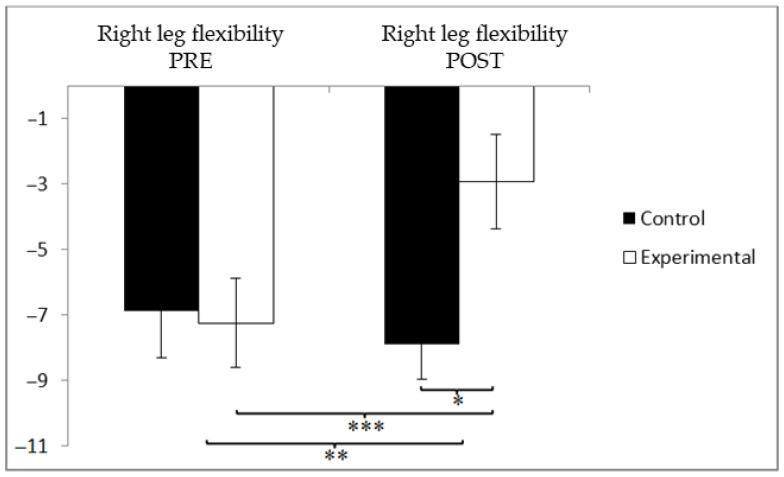
Between-group and within-group comparisons of right leg flexibility. * *p* < 0.05. ** *p* < 0.01. *** *p* < 0.001.

In the assessment of lower limb flexibility using the right leg, the participants in the experimental group (EG) initially recorded slightly better values (−7.81 ± 10.03) compared to those in the control group (CG), who averaged −6.56 ± 8.36. However, this trend was reversed following the intervention, with the CG showing greater improvement (−7.07 ± 9.09) than the EG (−2.53 ± 7.73). While both groups exhibited slight improvements in lower-limb flexibility, the experimental group demonstrated a more pronounced and statistically significant change, indicating a greater functional benefit associated with the intervention. Statistical analysis revealed a significant interaction between Group and Time, F(1, 90) = 14.732, *p* = 0.000, and η^2^ = 0.141, as well as a significant main effect for Time, F(1, 90) = 9.987, *p* = 0.002, and η^2^ = 0.100. No significant differences were found for the Group factor alone, F(1, 90) = 0.949, *p* = 0.333, and η^2^ = 0.010 (Figure 6). Detailed analysis of the interaction confirmed statistically significant post-intervention differences between groups, t(90) = −2.582 and *p* = 0.011, with a medium effect size (Cohen’s d = 0.54). Moreover, within the experimental group, significant improvements were observed from pre- to post-intervention, t(46) = −5.866 and *p* = 0.000, also reflecting a medium effect size (d = 0.59).

**Figure 7:**
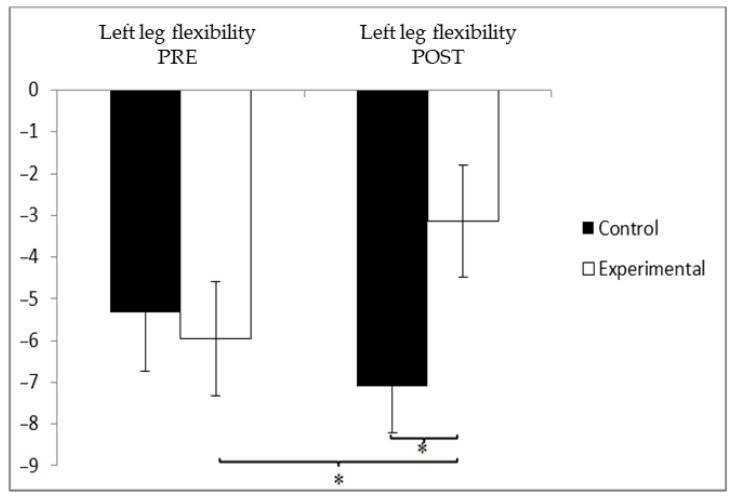
Between-group and within-group comparisons of left leg flexibility.* *p* < 0.05.

For lower limb flexibility in the left leg, baseline measurements showed that the participants in the experimental group (EG) had slightly better values (−6.66 ± 9.50) compared to those in the control group (CG), who recorded −5.13 ± 8.43. However, after the intervention, the CG exhibited greater improvement (−6.68 ± 8.85) than the EG (−3.09 ± 6.98). A statistically significant Group × Time interaction was observed, F(1, 90) = 16.690, *p* = 0.000, and η^2^ = 0.156. In contrast, no significant main effects were found for Group, F(1, 90) = 0.351, *p* = 0.555, and η^2^ = 0.004, or for Time, F(1, 90) = 3.006, *p* = 0.086, and η^2^ = 0.032 (Figure 7). Further analysis of the interaction indicated statistically significant differences between the two groups in the post-intervention measurements, t(90) = −2.107 and *p* = 0.038, with a small effect size (Cohen’s d = 0.45). Additionally, within the experimental group, significant differences were observed between pre- and post-intervention values, t(46) = −4.579 and *p* = 0.015, with a small effect size (d = 0.43).

### 3.4. Balance

**Figure 8:**
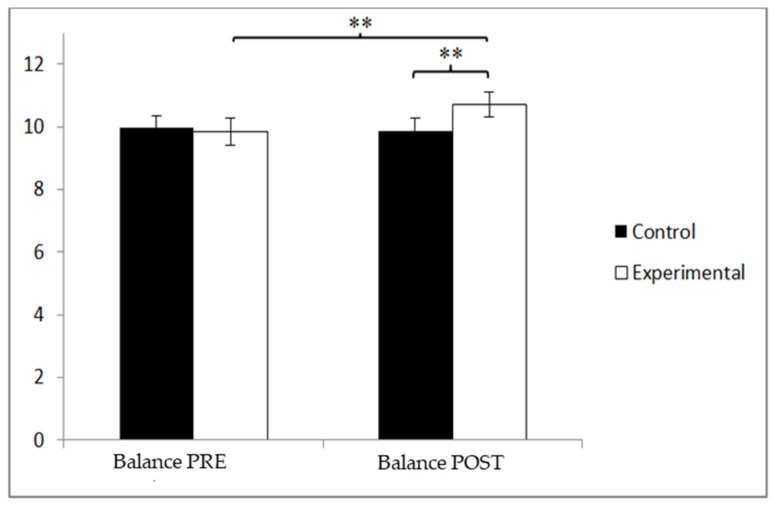
Inter- and intra-group comparisons for balance. ** *p* < 0.01.

In terms of balance, the participants in the control group (CG) initially reported slightly higher scores (10.04 ± 2.58) than those in the experimental group (EG), who had a mean of 9.85 ± 2.65. However, following the intervention, the EG demonstrated greater improvement, with a mean score of 11.26 ± 2.87, compared to the CG’s 9.71 ± 2.42. A significant interaction effect between Group and Time was observed, F(1, 90) = 10.423, *p* = 0.002, and η^2^ = 0.104. No statistically significant main effects were found for Time, F(1, 90) = 3.959, *p* = 0.050, and η^2^ = 0.042, or for Group, F(1, 90) = 1.982, *p* = 0.163, and η^2^ = 0.022 (Figure 8). Further examination of the interaction revealed statistically significant post-intervention differences between the two groups, t(90) = −2.785 and *p* = 0.007, with a medium effect size (Cohen’s d = 0.58). Moreover, within the experimental group, significant differences were found between pre- and post-intervention measurements, t(46) = −3.094 and *p* = 0.003, also with a medium effect size (d = 0.51). Although the Time effect approached the threshold for statistical significance (*p* = 0.050), this result may reflect inter-individual variability or the limited sensitivity of the balance subscore in detecting short-term changes. Notably, the observed improvement in the experimental group (Cohen’s d = 0.58) suggests a clinically meaningful gain, potentially contributing to enhanced postural stability and fall prevention in older adults with mild cognitive impairment.

**Figure 9:**
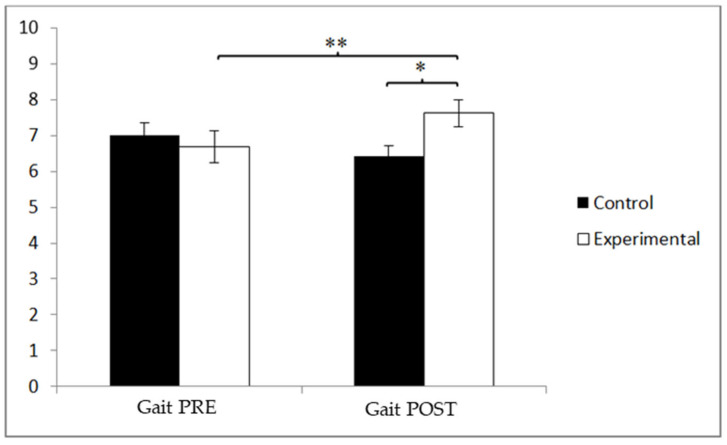
Inter- and intra-group comparisons for gait. * *p* < 0.05. ** *p* < 0.01.

In terms of gait, prior to the intervention, the participants in the control group (CG) showed higher scores (7.00 ± 2.61) compared to those in the experimental group (EG) (6.57 ± 2.32). However, following the intervention, the EG exhibited higher scores (7.60 ± 2.01) than the CG (6.71 ± 2.23). A significant interaction effect between Group and Time was found, as follows: F(1, 90) = 10.954, *p* = 0.001, and η^2^ = 0.109. No significant main effects were observed for Time: F(1, 90) = 3.423, *p* = 0.068, and η^2^ = 0.037, or for Group: F(1, 90) = 0.276, *p* = 0.601, and η^2^ = 0.003 (Figure 9). A detailed analysis of the interaction revealed statistically significant differences between the two groups in the post-intervention assessment, t(90) = −2.001 and *p* = 0.048, with a small effect size (d = 0.42). Additionally, significant improvements were observed within the EG when comparing pre- and post-intervention scores, t(46) = −3.530 and *p* = 0.001, also with a small effect size (d = 0.47). Despite the absence of a significant main effect for Time or Group, the improvement observed in the experimental group, reflected in a small but meaningful effect size (Cohen’s d = 0.42), suggests a practical enhancement in gait performance. This supports the intervention’s specific impact on mobility in older adults with mild cognitive impairment.

**Figure 10:**
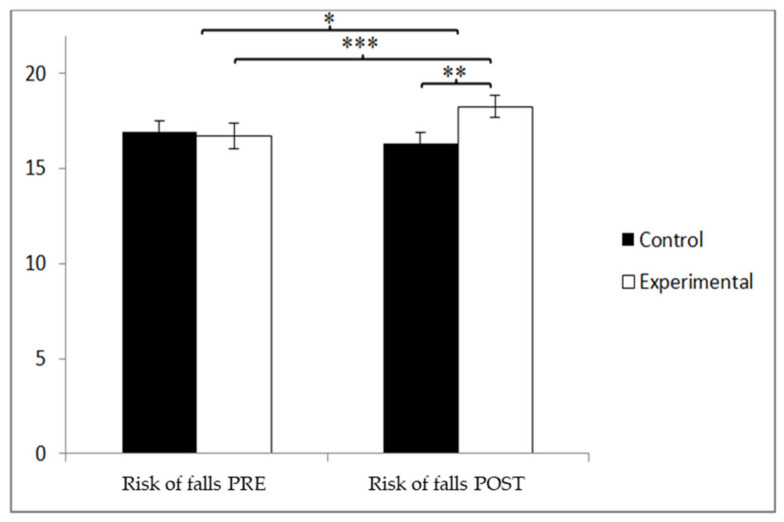
Inter- and intra-group comparisons for total score on the Tinetti scale. * *p* < 0.05. ** *p* < 0.01. *** *p* < 0.001.

Regarding the overall score on the Tinetti scale, which evaluates fall risk, the participants in the control group (CG) initially scored higher (17.04 ± 4.05) than those in the experimental group (EG) (16.43 ± 3.84) prior to the intervention. However, after the intervention, the EG achieved higher scores (18.85 ± 3.66) than the CG (16.42 ± 3.51). A significant Group × Time interaction was found, as follows: F(1, 90) = 16.342, *p* = 0.000, and η^2^ = 0.154, along with a significant main effect of Time: F(1, 90) = 5.721, *p* = 0.019, and η^2^ = 0.060. No significant main effect was observed for Group: F(1, 90) = 1.720, *p* = 0.193, and η^2^ = 0.019 (Figure 10). Further analysis of the interaction revealed statistically significant post-intervention differences between the groups, t(90) = −3.245 and *p* = 0.002, with a medium effect size (d = 0.67). Additionally, the EG showed significant improvement from pre- to post-intervention, t(46) = −3.860 and *p* = 0.000, also with a medium effect size (d = 0.65).

## 4. Discussion

The primary objective of this study was to evaluate the effectiveness of a 12-week dance-based aerobic training program on functional capacity and fall risk in older adults with mild cognitive impairment (MCI). Key variables, such as muscle strength, flexibility, balance, gait speed, and the total Tinetti score, were analyzed to determine the impact of the intervention. The results showed significant improvements in the experimental group (EG) compared to the control group (CG) in all variables analyzed. The most notable effects were observed in muscle strength, balance, and flexibility, which translated into a decreased risk of falls. This reduction is clinically relevant, as fewer falls are directly associated with a lower incidence of fractures, reduced hospital admissions, and preserved functional independence in older adults. The intervention was particularly effective in inducing functional improvements, with effect sizes ranging from small to moderate, and in some cases, large.

Muscle strength is a key pillar for maintaining functional independence in old age, as its decline is associated with an increased risk of falls, disability, and mortality [38]. In the present study, the participants in the experimental group (EG) showed a statistically significant increase in handgrip strength, from 17.22 ± 3.88 to 18.97 ± 3.56 kg after 12 weeks of the intervention. This improvement was not only significant compared to the baseline measurement but also compared to the control group (CG), with a high effect size (*p* < 0.001, d = 0.86), indicating a clinically relevant magnitude. This result is particularly valuable considering that the intervention did not use specialized equipment or resistance machines, but rather a modality based on choreographed dance, with functional movements performed in a rhythmic, continuous, and progressive manner. Unlike conventional strength training, which typically focuses on isolated contractions of specific muscle groups, this study’s approach integrated comprehensive movements that involve entire muscle chains through movements, twists, arm lifts, and repetitive sequences synchronized to music. This not only improves isometric and dynamic strength functionally, but also increases motivation and adherence to exercise. However, given that muscle strength tends to decline rapidly in older adults without continued physical activity, these gains may not be maintained long-term unless the intervention or similar training is sustained. For this reason, future studies should examine the long-term effects of dance-based interventions to determine the durability of their physical benefits over time. Previous studies, such as that by Chuang et al. [39], observed improvements in muscle strength in older adults with MCI through generalized aerobic programs, but the magnitude of the effects was smaller (d = 0.39), and the interventions did not incorporate motivational or creative components, such as music or dance. Furthermore, while most traditional programs are developed in clinical settings or with technical equipment, our intervention can be applied in community spaces with minimal resources, which favors its scalability. Another relevant differentiating factor is the target population. This study focused exclusively on older adults with mild cognitive impairment, a particularly vulnerable group underrepresented in clinical trials of strength training. Studies such as that by Labban et al. [40], for example, have reported benefits of resistance exercise in older adults without cognitive impairment, but did not address how the simultaneous integration of physical and cognitive components, as occurs in dance, can further enhance outcomes in individuals with MCI. Furthermore, studies have demonstrated improvements in muscle strength through interventions other than dance, such as Tai Chi or Pilates [41,42], but these activities often require highly trained instructors and longer motor learning times. In contrast, our dance program was accessible from the first sessions, facilitating participation even in subjects with mild cognitive impairment. Furthermore, unlike other modalities, such as Pilates or Tai Chi, dance-based interventions inherently combine physical movement with rhythm, memory, sequencing, and coordination, offering a unique dual-action benefit that targets both motor and cognitive domains in individuals with mild cognitive impairment (MCI).

Gait speed is a robust clinical marker, used as a prognostic indicator of frailty, functional dependence, hospitalization, and even mortality in older adults [43]. Values below 1 m/s have been consistently associated with an increased risk of physical and cognitive decline, so small improvements in this variable can have a substantial clinical impact [44]. In our study, the experimental group (EG) showed a significant improvement in gait speed, from 0.94 ± 0.38 to 1.01 ± 0.17 m/s after 12 weeks of the intervention, while the control group (CG) showed no significant changes (0.96 ± 0.13 to 0.94 ± 0.12 m/s). This difference was not only statistically significant (*p* < 0.05) but also reached a clinically relevant effect size (d = 0.48), indicating real functional improvement in this vulnerable population. Although previous studies, such as that by Trombetti et al. [45], observed similar benefits following musical multitasking training interventions (which included walking to music and performing simultaneous cognitive tasks), our protocol differed in several key aspects. First, while Trombetti et al. applied their intervention three times a week for six months, our study achieved comparable results with only two weekly sessions for 12 weeks, demonstrating the greater effectiveness of the dance-only program. Second, our intervention focused on older adults with mild cognitive impairment (MCI), a population at higher risk for gait disturbances and falls, while most similar studies include samples with intact or heterogeneous cognitive function. Another differentiating element was the progressive structure of the choreographies, carefully designed to challenge the participants’ physical and cognitive abilities. The incorporation of multidirectional movements, turns, rhythm changes, and complex motor patterns, accompanied by constant musical stimuli, may have facilitated a more effective integration between sensory, motor, and cognitive systems. This simultaneous activation of brain networks has been associated with improvements in motor control and processing speed, key elements for maintaining a stable and efficient gait. Recent studies, such as that by Rehfeld et al. [46], have shown that dance stimulates brain regions involved in balance and locomotion, in addition to promoting neuroplasticity even in older adults, reinforcing the neurophysiological plausibility of our findings. Furthermore, when compared with more conventional interventions focused on walking or strengthening exercises, such as those reviewed by Sherrington et al. [47], the effects on gait speed are generally modest (increases of 0.03–0.05 m/s) and require longer training duration or intensity. Our study, in contrast, obtained a clinically significant improvement with a reduced weekly dose and a more engaging and motivating modality for participants. This difference suggests that the cognitive–affective component and multisensory nature of dance may enhance the physical effects of training more efficiently than traditional methods. Although cognitive outcomes were not measured in this study, the neurophysiological rationale for dance-based interventions suggests a potential cognitive benefit, particularly in individuals with MCI, that warrants exploration in future research.

Flexibility is a fundamental physical ability that tends to deteriorate with age, directly affecting joint mobility, the efficiency of daily movements, and the ability to react to imbalances, which increases the risk of falls and functional dependence [48]. In our study, the experimental group (EG) showed significant improvements in flexibility in both the upper and lower extremities. In particular, a high effect size was observed for the flexibility of the left arm (d = 0.79), and moderate effects were observed for other measures, such as the right arm (d = 0.50) and legs (d = 0.43–0.59), demonstrating a generalized functional gain. Notably, the observed improvements in flexibility were achieved without the implementation of specific stretching protocols, underscoring the effectiveness of dance-based routines in promoting joint mobility and range of motion through dynamic and rhythmic movement. These results are even more significant considering that the improvements were achieved not through a flexibility-specific intervention, but rather through a choreographed dance activity. This suggests that the repetitive, broad, and rhythmic movements of dance routines induce active and dynamic elongation of large muscle groups and improve joint mobility. Unlike passive stretching exercises, dance incorporates flexibility within a functional and natural context, facilitating its transfer to activities of daily living. Previous research has obtained similar results, such as Federici et al. [49], who observed similar benefits in older adults through choreographic training. However, their study did not include individuals with cognitive impairment or assess flexibility separately by body segment, as we did in our protocol with the back scratch and chair sit-and-reach tests, which provides a greater level of functional precision to our findings. Furthermore, our intervention lasted 12 weeks, longer than the 8–10 weeks commonly reported in similar programs, which may have allowed for a progressive consolidation of physiological changes. Cruz-Ferreira et al.’s [50] study, which used creative dance programs in healthy older adults, also reported improvements in flexibility; however, their results focused on general mobility measures, without specifying segments or using standardized instruments. Furthermore, their sample did not include individuals with MCI, who tend to present with greater joint stiffness and motor limitations related to neuromuscular impairment. On the other hand, research that has addressed flexibility through more traditional methods, such as yoga or Pilates [51,52], has also reported benefits, but requires levels of concentration, balance, and body control that can represent a barrier to entry for individuals with cognitive impairment. In this sense, our intervention approach, musically and choreographically adapted to the functional level of the participants, represents a more accessible, motivating, and safe alternative for effectively working on flexibility in older adults with MCI. Furthermore, dance offers a cognitively accessible format that can be easily adapted to different levels of cognitive functioning. Unlike modalities such as yoga or Pilates, dance does not rely heavily on sustained attention or complex motor planning, making it a more inclusive intervention for older adults with MCI.

Balance is a complex functional ability that integrates sensory information, central processing, and coordinated motor responses [53]. Its impairment is common in older adults, especially those with MCI, who present with impairments in both postural control and motor planning [54]. In this study, the experimental group (EG) showed significant improvements in balance on both the balance and gait subscales of the Tinetti scale, with an average total increase of more than 2 points compared to the control group. This improvement represents a clinically relevant change, as differences of 1–3 points on this scale are associated with reductions in the risk of falls and greater functional stability. These findings partially coincide with the results of Kattenstroth et al. [55], who observed improvements in postural control after a dance-based intervention. However, their sample consisted of older adults without cognitive impairments, which limits the extrapolation of their results to populations with MCI. Furthermore, studies such as Hackney et al. [56], which used adapted tango as an intervention in older adults with Parkinson’s, also reported improvements in balance.

The risk of falls represents one of the main challenges in geriatric care, especially in people with MCI, who present impairments in both motor function and the executive processing required to respond to unexpected events during gait [57]. In our study, the EG showed a significant improvement in the total Tinetti scale score, increasing from an average of 16.43 ± 3.84 to 18.85 ± 3.66, while the CG showed no changes. The moderate effect sizes observed both within (d = 0.54) and between (d = 0.62) groups indicate not only statistical significance but also clinical relevance. Notably, the mean score in the experimental group increased sufficiently to move participants from a borderline risk category to a lower risk zone for falls, suggesting a substantial functional benefit from the intervention. Furthermore, a change of more than 2 points on the Tinetti balance subscale is clinically meaningful, as it brings participants closer to the threshold of low fall risk, thereby reinforcing the functional relevance of the observed improvements. The effect size was moderate–high (d = 0.67), indicating a clinically relevant reduction in the risk of falls after the intervention. These results are particularly significant, as the Tinetti scale is a widely validated tool that allows for a comprehensive estimation of falls risk by assessing balance and gait. Unlike studies, such as that by Merom et al. [57], who reported a reduction in the incidence of falls through community dance programs, our controlled design and use of standardized measures allow for a more robust association between the intervention and the observed functional changes. Furthermore, while in their study, the frequency and intensity of the sessions were variable and poorly structured, our intervention was delivered in a consistent manner, with a specific dosage (two sessions per week for 12 weeks) and a technical progression designed to stimulate balance, strength, coordination, and motor memory. Furthermore, the multicomponent nature of our program, which combined rhythmic movements, variable-amplitude movements, weight transfer, postural changes, and sequential memory training, favored the integration of different physical abilities into a single format, thus facilitating functional transfer to real-life situations, such as turning, braking suddenly, or maintaining balance while climbing stairs.

It is also important to consider the gender distribution of the sample when interpreting the findings. In this study, women represented 60.4% of the total participants. Previous research suggests that older women are not only more likely to volunteer for community-based physical activity programs but may also exhibit higher coordination, rhythmic ability, and adherence in dance-based interventions compared to men. These factors could have contributed to the favorable outcomes observed, particularly in variables such as balance, gait speed, and flexibility. While the gender distribution reflects common patterns in real-world program participation, future studies should aim for more balanced samples to explore potential sex-based differences in response to dance-based training.

This study has some limitations that should be considered. First, although the sample was statistically adequate, its size limits the extrapolation of the findings to larger populations or those with more advanced levels of cognitive impairment. Second, the follow-up duration was limited to 12 weeks; longitudinal evaluations would be relevant to determine the sustainability of the benefits. Third, while gait speed is a well-established indicator of functional mobility and fall risk in older adults, it may not capture the full complexity of the motor and cognitive interaction. Future studies could benefit from including additional measures of executive function or dual-task mobility to provide a more comprehensive assessment of functional outcomes. No adverse events related to the intervention were reported, and participant dropouts were not attributed to safety concerns, reinforcing the tolerability and feasibility of the program.

## 5. Conclusions

The results of this study suggest that a structured aerobic dance-based training program, tailored to older adults with mild cognitive impairment and performed at moderate intensity, may lead to improvements in various dimensions of functional capacity—including muscle strength, flexibility, balance, and gait speed—as well as a potential reduction in fall risk. While the observed changes are encouraging, they should be interpreted in light of the specific characteristics of the intervention (e.g., choreographed line dancing, familiar music, and progressive complexity) and the demographic profile of the participants. The intervention, characterized by its playful, accessible, and low-cost nature, was well-tolerated and achieved high adherence, even at a moderate frequency and without the use of specialized equipment, factors that support its feasibility in community-based contexts, such as senior centers or nursing homes. Unlike approaches targeting isolated physical functions, the choreographed, multicomponent format of dance may offer simultaneous cognitive and motor engagement, potentially enhancing the transfer to daily functional tasks. These findings contribute to the growing body of literature on dance as a promising non-pharmacological strategy for promoting autonomy and fall prevention in older adults with MCI. However, further research, especially with longitudinal designs, balanced gender samples, and neurocognitive outcome measures, is needed to better understand the long-term effects and generalizability of such interventions.

## Figures and Tables

**Table 1:** Baseline sociodemographic and clinical profile of participants: total sample and group comparison.

		Total (n = 92)	Experimental (n = 47)	Control (n = 45)	Value *p*
Age		71.83 ± 2.96	71.43 ± 2.97	72.24 ± 2.92	0.783
Sex	Male	34 (23.10)	18 (52.90)	16 (47.10)	0.672
	Female	58 (39.50)	29 (50.00)	29 (50.00)	
Occupational Status	Retired	67 (45.60)	35 (52.20)	32 (47.80)	0.586
	Working	0 (0.00)	0 (0.00)	0 (0.00)	
	Unemployed	25 (17.00)	12 (48.00)	13 (52.00)	
Marital Status	Single	13 (8.80)	7 (53.80)	6 (46.20)	0.710
	Married	52 (35.40)	26 (50.00)	26 (50.00)	
	Divorced/Separated/Widowed	27 (18.40)	14 (51.90)	13 (48.10)	
Educational Status	No Education	14 (9.50)	8 (57.10)	6 (42.90)	0.090
	Primary Education	54 (36.70)	31 (57.40)	23 (42.60)	
	Secondary Education	16 (10.90)	5 (31.20)	11 (68.80)	
	University Education	8 (5.40)	3 (37.50)	5 (62.50)	
Height		1.66 ± 0.13	1.66 ± 0.13	1.65 ± 0.12	0.965
Weight		73.36 ± 11.95	72.57 ± 11.42	74.18 ± 12.56	0.984
BMI		26.99 ± 5.06	26.42 ± 4.79	27.60 ± 5.32	0.758
Waist Circumference		95.79 ± 9.00	93.94 ± 8.90	95.68 ± 9.13	0.798
Hip Circumference		107.27 ± 7.41	106.62 ± 7.17	107.95 ± 7.68	0.606
Waist–hip Ratio		0.89 ± 0.63	0.88 ± 0.68	0.89 ± 0.55	0.111

Quantitative variables are presented as mean and standard deviation. Qualitative variables are presented as frequency and percentage. BMI: Body Mass Index.

## Data Availability

The data presented in this study are available upon request from the corresponding author. The data are not publicly available because, due to the sensitive nature of the questions asked in this study, the participants were assured that raw data would remain confidential and would not be shared.
